# Revisiting HER2 in Prostate Cancer from an Inclusive Perspective: From Biomarkers to Omics

**DOI:** 10.3390/cancers16193262

**Published:** 2024-09-25

**Authors:** Nicole Mavingire, Janelle C. Moore, Jabril R. Johnson, Abdulrahman M. Dwead, Cheryl D. Cropp, Yehia Mechref, Firas Kobeissy, Soroush Rais-Bahrami, Leanne Woods-Burnham

**Affiliations:** 1 Department of Surgery, Morehouse School of Medicine, Atlanta, GA 30310, USA; nmavingire@msm.edu (N.M.); jcmoore@msm.edu (J.C.M.);; 2 Department of Microbiology, Biochemistry, & Immunology, Morehouse School of Medicine, Atlanta, GA 30310, USA; jabjohnson@msm.edu; 3 Department of Pharmacology & Toxicology, Morehouse School of Medicine, Atlanta, GA 30310, USA; ccropp@msm.edu; 4 Department of Chemistry and Biochemistry, Texas Tech University, Lubbock, TX 79409, USA; 5 Department of Neurobiology, Morehouse School of Medicine, Atlanta, GA 30310, USA; fkobaissy@msm.edu; 6 Department of Urology, University of Alabama at Birmingham Heersink School of Medicine, Birmingham, AL 35294, USA; sraisbahrami@uabmc.edu; 7 Department of Radiology, University of Alabama at Birmingham Heersink School of Medicine, Birmingham, AL 35294, USA; 8 O’Neal Comprehensive Cancer Center, University of Alabama at Birmingham Heersink School of Medicine, Birmingham, AL 35294, USA

**Keywords:** HER2, *ERBB2*, prostate cancer, Black men, genetic ancestry, omics

## Abstract

**Simple Summary:**

HER2 is a well-known driver of worse outcomes for many types of cancer, including prostate cancer. While HER2 has been previously evaluated in prostate cancer bench studies and clinical trials, there has been a lack of diversity in the experimental designs and protocols. For this reason, it has only recently been reported that Black men with prostate cancer may have a higher prevalence of HER2 overexpression. To thoroughly address health inequities that exist for Black men with prostate cancer, it is critical to utilize diverse biospecimens and enroll diverse study participants into studies that evaluate genetic and molecular contributors to worse prognosis for high-risk populations. In this review, we reconsider the role of HER2 in prostate cancer with an approach that incorporates the effects of race and genetic ancestry.

**Abstract:**

Human epidermal growth factor receptor 2 (HER2) is a major driver of disease progression, treatment resistance, and worse survival for patients with various types of cancers, including prostate cancer. However, key bench studies and clinical trials have failed to evaluate the role of HER2 in prostate cancer using racially diverse experimental designs and protocols. This lack of diversity represents what has been the status quo of cancer research in the United States for decades. In the case of prostate cancer, homogenic study designs are problematic as Black men are much more likely to be diagnosed and die from aggressive and incurable forms of the disease. Therefore, the strategic inclusion of biospecimens collected from Black patients as well as the recruitment and enrollment of Black men into prostate cancer clinical trials is necessary to comprehensively evaluate genetic and molecular factors that contribute to variable outcomes in this high-risk population. Additionally, a higher prevalence of HER2 expression in Black men was recently reported in a small cohort of prostate cancer patients and may contribute to worsened prognosis. In this review, we carefully consider the role of HER2 in prostate cancer while, for the first time, taking into account the influences of race and genetic ancestry.

## 1. Introduction

Globally, prostate cancer (PC) is the most frequently diagnosed cancer in men, after adjusting for age, followed by lung cancer [1]. PC is the second-deadliest cancer impacting men and the fifth-most fatal cancer in the world [1]. The burden of disease globally is substantial, but the more developed nations tend to have higher incidence rates while developing countries tend to have higher mortality rates than the developed nations [1,2]. It is estimated that by 2040, PC incidence will more than double to 9 million cases diagnosed annually, worldwide, mostly due to increased life expectancy and changes to other demographics [2]. Late diagnosis is widespread worldwide and is often tied to limited treatment options for patients and higher mortality rates, particularly in less developed nations [1,2]. While many PC therapies are now considered affordable, equitable distribution and access to care remain a serious global problem [2]. In contrast, early diagnosis significantly improves patient prognosis and survival outcomes, while reducing the societal and individual cost [2]. However, a severe disparity exists in that men of African descent (Black) experience more than twice the incidence and mortality rates of men of European (white) origin [2,3]. We need to better understand the drivers of the observed ethnic differences in incidence and yet, most PC research is disproportionately focused on white men. Given that therapeutic decision making, clinical trial design, and outcomes in PC are heavily contingent upon accurately stratifying patients into risk groups (e.g., low-risk vs. high-risk) [4], it is crucial to distinguish between indolent and more aggressive forms of the disease by identifying novel druggable biomarkers [5,6].

In 2024, an estimated 299,010 new PC diagnoses will be made in the United States (U.S.) and 35,250 men will die from this disease [3]. Black men deal with an extraordinary PC burden, with incidence rates 68% higher than white men, two times higher than American Indian and Alaska Native and Hispanic men, and three times higher than Asian American Pacific Islander men [3]. Black men also experience the highest disease-specific mortality rates compared to other ethnicities in the U.S. [3]. Fluctuations in prostate-specific antigen (PSA) screening recommendations have partly contributed to this disparity. PSA is the most common biomarker used in PC screening and diagnosis in combination with determination of PC staging and Gleason scoring [2,3]. The United States Preventive Services Task Force (USPSTF) recommends a PSA cut-off threshold of 4 ng/mL for PC screening and a follow-up prostate biopsy when PSA levels > 4.0 ng/mL are detected [7]. However, clinically significant PC can be missed in some patients based on >4 ng/mL PSA levels. Furthermore, the USPSTF recommends screening discussions to begin at the age of 55, which misses men who could be diagnosed as early as 40 [7]. We recently noted that the data used to draw these recommendations were based on populations of primarily European descent and the data are in opposition to global evidence of the increased PC incidence and mortality in Black men and to the detriment of these high-risk populations over time [7]. Granted, the low specificity and high sensitivity of PSA to respond to benign stimuli limit the value of PSA as a PC therapeutic target [5]. For this reason, additional genes, biomarkers, and tests have been identified and tested in predominantly white populations, but there remains a need for distribution and testing in less developed nations that include predominantly Black populations [2,5].

Potentially, reevaluating currently targeted biomarkers in other cancers could provide much-needed additions to the PC diagnosis and treatment pathways at a quicker pace than the discovery of new markers. We believe that the human epidermal growth factor receptor 2 (HER2) is one such marker. HER2 is most known for its successful utility in breast cancer (BC) as a diagnostic tool and therapeutic target [8]. HER2 is also a well-known driver of worse outcomes for many types of cancer, including PC. For example, HER2 overexpression has been linked to rapid prostate tumor growth and poor patient prognosis in multiple studies [9,10,11,12]. It has only recently been reported that Black PC patients may have a higher prevalence of HER2 overexpression [13]. In this review, we explore the role of HER2 as a potential PC biomarker and therapeutic target for the high-risk Black population. We discuss the role of HER2 in a range of diseases, including BC, before focusing on PC in depth. We provide and discuss multiple bioinformatics analyses of diverse datasets that show the many regulatory interactions, ubiquitous role, and crucial importance of HER2 as a biomarker within many diseases including PC. We also evaluate the role of genetic ancestry in the etiology of PC, the current state of ethnically/racially diverse biospecimens and preclinical models for PC research, as well as the lack of diversity in clinical trials. Our long-term goal is to reduce the disparities in PC prognosis, treatment outcomes, and mortality for high-risk groups.

## 2. HER2 in Cancer

HER2 is a member of the class I receptor tyrosine kinase family and it shares substantial homology with epidermal growth factor receptor (EGFR), HER3, and HER4 [8,14]. HER2 protein is expressed in normal and malignant epithelial cells and plays roles in regulating cell proliferation, survival, and differentiation [15]. The dimerization of HER2 receptors initiates intracellular signaling pathways in response to described interactions with external signaling molecules known as growth factors as well as other mechanisms that remain to be investigated. These pathways orchestrate controlled cell growth and embryonic development [15]. However, cellular dysregulation stemming from HER2 mutations or overexpression can prompt carcinogenesis [16]. Overexpression and mutations of HER2 have been identified across a spectrum of solid organ malignancies spanning breast, colorectal, bladder, gastric, esophageal, endometrial, and ovarian cancers and numerous other diseases.

**Figure 1:**
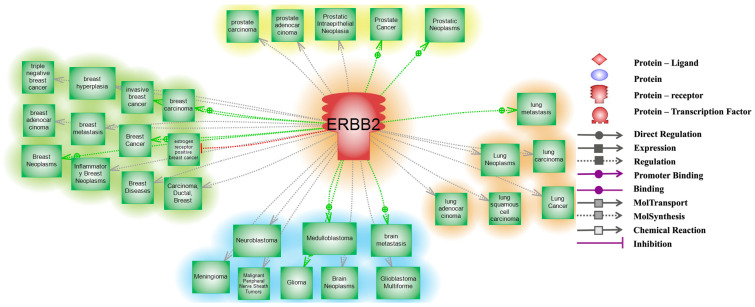
Erythroblastic oncogene B receptor tyrosine kinase (*ERBB2*)/Human epidermal growth factor receptor 2 (HER2) role in different types of cancers. A schematic diagram depicting the diverse prominent role of HER2 in different kinds of cancers involving lung, brain, breast, and prostate cancer where *ERBB2*/HER2 plays a major role in different levels of cancer genesis, development, and metastasis. Please refer to the supplementary data for detailed descriptions of the role of HER2 shown here. Pathway Studio^®^ powered by Elsevier was used for this analysis [17].

**Figure 2:**
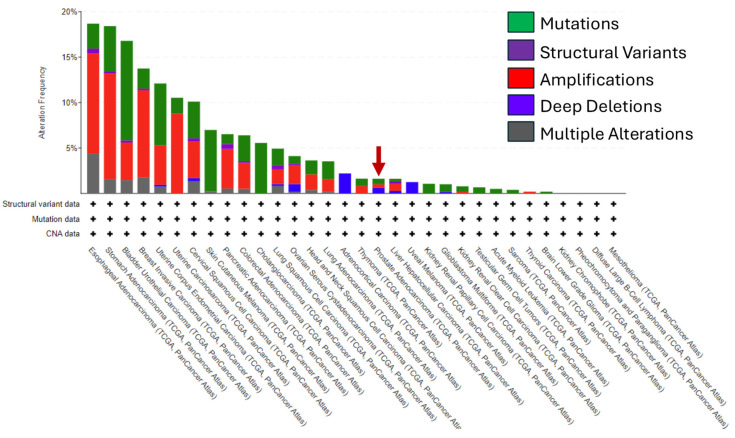
A pan-cancer analysis of genetic alterations to the HER2 gene (*ERBB2*). A representative bar graph shows the relatively low frequency of genetic alterations in *ERBB2* in prostate cancer compared to other cancers. The data used for this analysis were accessed via The Cancer Genome Atlas (TCGA) on the Genomic Data Commons Data Portal website accessed on 10 September 2024 (https://portal.gdc.cancer.gov/) [54].

**Figure 3:**
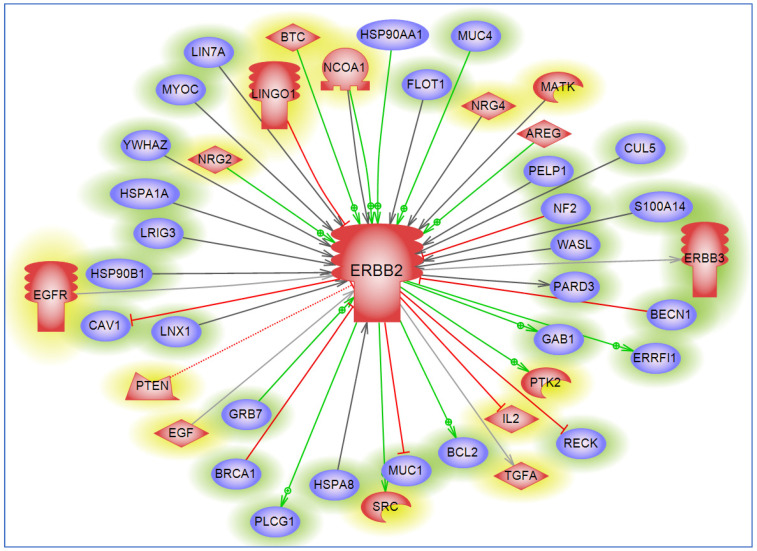
ERBB2 protein (HER2) regulates over 50 proteins within different diseases. A schematic shows our bioinformatics analysis that identified 56 proteins downstream of ERBB2/HER2 that are either regulated by direct binding or modulated by post-translational modification (PTM) or other regulatory pathways detailed in the supplementary data. These interactions depict the ubiquitous regulatory role of ERBB2 on several sets of proteins/transcription factors; these are implicated in several diseases as shown in Figure 1. Pathway Studio^®^ powered by Elsevier was used for this analysis [17]. Please refer to the interaction entity Legend table in Figure 1.

**Figure 4:**
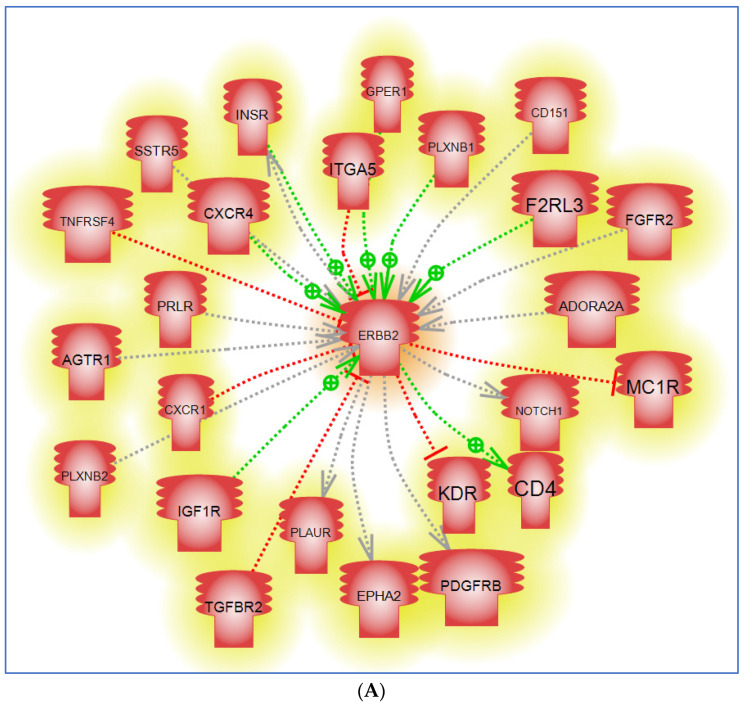

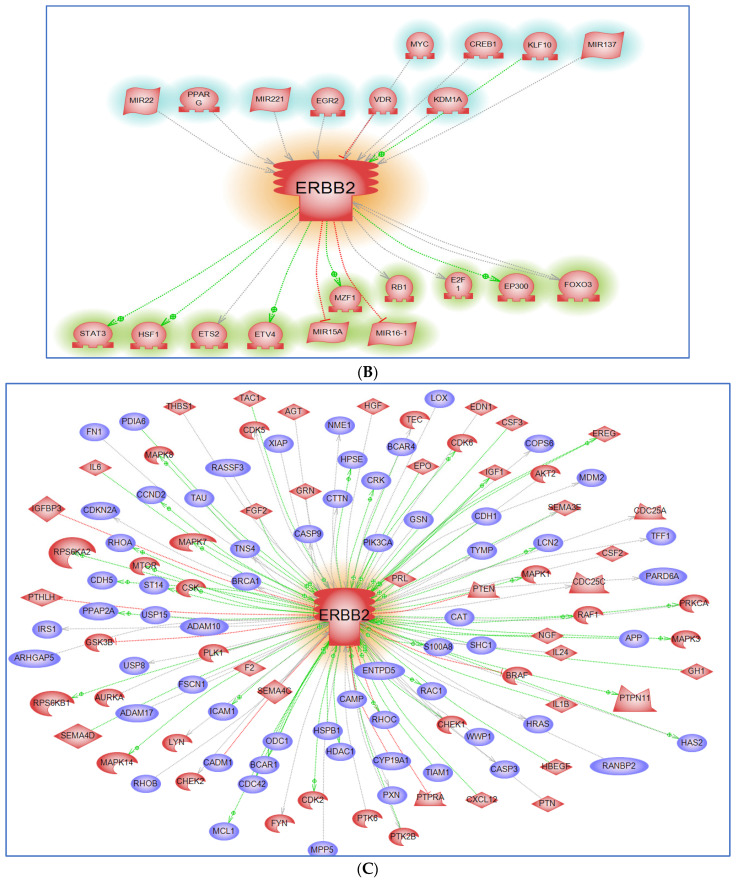
An *ERBB2*/HER2 “Direct” protein–protein interactome analysis map. This schematic depicts the diverse protein interactions of HER2 with other different protein markers including receptors (**A**), transcription factors and microRNA (**B**), and specific protein families (**C**). More than 150 protein interactions with HER2 involving positive regulation, inhibition, binding, PTM regulation, translation, or modulation are highlighted. Please refer to the supplementary data for detailed descriptions of the interactions shown here. Pathway Studio^®^ powered by Elsevier was used for this analysis [17]. Please refer to the interaction entity Legend table in Figure 1.

To understand the potential role of HER2 in regulating several proteins within various disease states and within the cell, we performed interaction analyses using the PathwayStudio^®^ software (Elsevier Inc., Amsterdam, The Netherlands, https://www.elsevier.com/solutions/pathway-studio-biological-research) V:11 accessed on 10 September 2024 [17,18]. The latter consists of propriety-curated databases established to serve as a predictive functional annotation tool. To determine significantly altered functional and biological pathways for each set, the Subnetwork Enrichment Analysis algorithm was utilized. This algorithm uses Fisher’s exact test for the detection of nonrandom associations between two categorical variables that are organized by a specific relationship. In this regard, we interrogated the downstream disease targets that are associated or linked to HER2/erythroblastic oncogene B receptor tyrosine kinase 2 (*ERBB2)* (shown in Figure 1 and Supplementary Data S1), and we evaluated the frequency of genetic alterations of *ERBB2* in PC compared to other cancers (Figure 2). We also evaluated protein targets that are regulated by HER2/*ERBB2* (Figure 3, and our final enrichment analysis focused on identifying protein–protein interactions involved with HER2/*ERBB2* (Figure 4A–C). Raw data related to each individual-related regulation or interaction are provided in the supplementary data pertaining to each figure (Supplementary Data S2). Our analysis identified over 1700 roles of HER2/*ERBB2* in a wide range of diseases from brain injury/dysfunction to lymphomas, neoplasms, and carcinomas (Figure 1). The data show that HER2/*ERBB2* is a significant player and a hub regulator within multiple conditions including PC.

The events that occur in HER2-overexpressing cancers to promote disease progression, treatment resistance, and patient death have been delineated over forty years of combined research [17]. The presence or absence of HER2 overexpression currently steers subsequent treatment strategies and associated outcomes for multiple cancers but it began with BC. A diagnosis of highly aggressive HER2-positive (HER2+) BC was previously deemed incurable irrespective of the treatment modality selected [19,20,21]. Black and Hispanic women are more commonly diagnosed with HER2+ BC [22,23]. Determining HER2 status has been crucial for patients diagnosed with invasive BC, usually through multiple assessments. Initially, immunohistochemistry (IHC) is performed using a validated assay to evaluate HER2 protein expression [24]. IHC scoring is based on the following pattern of membrane staining: a score of 3 indicates positive HER2 expression, marked by intense staining of the cell membrane in over 30% of invasive tumor cells; a score of 2 indicates equivocal HER2 protein expression, where membrane staining is complete but may be uneven or faint in intensity, covering at least 10% of cells; and a score of 0 or 1 indicates negative HER2 protein expression, showing no significant staining of the cell membrane. Tissue specimens with inconclusive IHC results undergo confirmation through fluorescence in situ hybridization (FISH). FISH analysis quantifies the *ERBB2*-to-*CEP17* ratio and the count of *ERBB2* gene copies. Positive *ERBB2* amplification is indicated by a FISH ratio exceeding 2.2 or an *ERBB2* gene copy number surpassing 6.0. Equivocal *ERBB2* amplification is characterized by a FISH ratio ranging from 1.8 to 2.2 or an *ERBB2* gene copy number between 4.0 and 6.0. Negative *ERBB2* amplification is identified by a FISH ratio below 1.8 or an *ERBB2* gene copy number under 4.0. These methodologies ensure a precise assessment of HER2 status, thereby informing optimal treatment strategies in BC care [25].

The significantly diminished survival rate of BC patients with HER2+ disease ultimately spurred the production of the monoclonal antibody targeting HER2, trastuzumab [26]. The groundbreaking targeting of HER2 as a viable and remarkably responsive therapeutic option marked a pivotal advancement in addressing the aggressive nature of HER2+ BC. The development of subsequent anti-HER2 targeting drugs for clinical implementation has resulted in significantly enhanced survival rates for patients with varying degrees of HER2 staining intensity [27]. Furthermore, extensive translational research investigating HER2 as a druggable target has resulted in the generation of multiple Food and Drug Administration (FDA)-approved therapeutics in multiple cancers including lung, gastrointestinal, and BC [8,28,29]. Since the creation and approval of the HER2 antibody trastuzumab for BC treatment in the 1990s, there has been an eruption of HER2- and receptor tyrosine kinase (RTK)-targeted therapeutics that include monoclonal antibodies, antibody–drug conjugates, and receptor tyrosine kinase inhibitors [8,30]. However, these therapeutics are yet to be added to the PC treatment continuum.

## 3. HER2 and Genetic Ancestry

The use of genetic ancestry in biomedical research has become essential among research experts who understand that self-reported race is not a strong proxy for biology [31,32]. This is especially true when deciphering the role of West African genetic ancestry in studies that include Black populations in the U.S. The Black population in the U.S. is genetically heterogeneous due to historical influences [33], and the high level of variation observed in the population is due to the antiquity of the African gene pool and gene flow from non-African-descent populations [34,35]. Because of the heterogeneity in the genetic background of African-descent populations, genetic ancestry must be considered when studying populations throughout the diaspora. However, it is important to note that the majority of Black individuals in the U.S. derive their African ancestry from ancestors who are traced to various geographic regions of West and West–Central Africa, ranging from Senegal to Nigeria to Angola [36]. When comparing African admixture estimates to highly detailed census based on historical records, 95% of Africans were deported to the Americas largely from West–Central Africa, followed by West Africa [36,37,38,39]. Due to the routes of the trans-Atlantic slave trade, the average Black person in the U.S. carries approximately 80–90% West African genetic ancestry [36].

Variation in genetic risk factors across ethnic groups is increasingly recognized as one of several potential explanatory factors that may be associated with cancer health disparities including PC [22,40]. In the case of BC, risk and patient prognosis differ by tumor subtypes defined by category based on the presence or absence of estrogen receptor, progesterone receptor, and HER2 expression. White women have a higher prevalence of luminal A disease while Black and Hispanic women have a higher prevalence of basal-like and HER2 subtypes [22,23,41]. Although ethnic differences remain clinically evident, most genomics studies attempting to elucidate the underpinnings of this genetic heterogeneity have been conducted in populations of European descent [42]. Recently, progressive steps have been taken to better understand these differences based on genetic ancestry and social determinants of health in underrepresented ethnically diverse populations [43,44,45,46].

The impact of HER2 on cancer health disparities outside of BC has also been investigated. In an investigation of PC health disparities, gene set enrichment analysis showed oncogenic gene signatures, including *KRAS* and *ERBB2*, were enriched in patients expressing high *KRT15* and *KRT19* associated with poor androgen deprivation therapy (ADT) response after radical prostatectomy. These signatures demonstrated a higher expression in Black PC patients compared to PC patients of European ancestry [47]. Notably, HER2 overexpression in PC has only recently been evaluated in Black men by our team and one other group [13], even though gene expression profiles confirm biological differences in tumors from Black PC patients [48,49,50,51]. Since Black men are considerably more likely to be diagnosed with PC and die from the disease [4], there is a critical need to thoroughly evaluate the mechanisms and signaling pathways, including HER2, that drive PC progression and metastasis, leading to lethal outcomes in Black men. A comprehensive evaluation requires, primarily, the inclusion of Black men and secondly, the consideration of race and genetic ancestry as impactful determinants of disease manifestation and treatment response.

## 4. HER2 in Prostate Cancer

The role of HER2 in the oncogenesis and progression of PC remains poorly understood and controversial. Several studies have reported no link between HER2 expression and PC tumor grade (Gleason score), tumor/node/metastasis (TNM) stage, or patient prognosis [52,53,54,55,56,57]. In one study, only 1 out of 126 androgen-dependent, or -independent primary, and metastatic PC tumors contained borderline (6 to 8 copies) amplifications of *ERBB2* [52]. In a tissue microarray protein and gene expression analysis of 74 PC tumors, HER2 overexpression was observed in 73% of samples while gene amplification was present in only 4%. There was no relationship between HER2 protein expression or gene amplification and known prognostic parameters [53]. FISH analysis of 371 tissue samples that included benign prostatic hyperplasia (BPH), primary, locally recurrent, and metastatic PC showed no *ERBB2* amplifications in any PC samples irrespective of disease stage [54]. An IHC analysis of 53 primary PC and 9 BPH tissue samples showed that 33% of PC samples expressed HER2 without gene amplification as measured by polymerase chain reaction and 36-month patient follow-up revealed no correlation with disease progression [56]. In another study, an IHC analysis of 35 BPH, prostate intraepithelial neoplasia (PIN), and PC samples found greater HER2 expression in BPH and PIN than in PC samples [55].

Few studies have reported promising but inconclusive data on HER2 expression and its prognostic value in PC [57,58,59]. When Reese and colleagues used IHC and FISH analyses to evaluate HER2 expression and gene amplification in 39 androgen-independent PC samples, they found that 36% expressed HER2 but only 5% had either moderate expression (2+) or high-level expression (3+). Furthermore, only 6% of the samples had gene amplification. Unfortunately, the authors did not investigate potential correlations with known prognostic parameters [59]. An IHC analysis of 216 primary PC tissue samples reported weak focal staining representing HER2 expression in only 15% of samples; however, 97% of these samples had a Gleason score of 7 or greater [57]. While FISH analysis of 39 primary PC samples and 10 BPH samples showed greater HER2 overexpression in localized PC compared to BPH, no correlation with Gleason grade could be determined; thus, the research group reported that the prognostic value of HER2 remains unclear [58].

However, HER2 overexpression has been linked to rapid prostate tumor growth and poor patient prognosis in multiple other studies [10,11,12,60]. A large study analyzing over 2500 PC tissue samples in a microarray using IHC and FISH showed weak HER2 staining (<2+) in approximately 20% of samples and *ERBB2* gene amplification in one sample; however, significant associations were found between positive staining and known prognostic parameters (high Gleason grade, advanced TNM stage, rapid tumor cell proliferation, and tumor recurrence) [9]. An IHC and FISH analysis of HER2 protein expression and gene expression in 252 primary PC patients with long-term follow-up found significant associations between protein and gene expression as the disease progressed (Gleason score), metastasized (lymph node involvement), and led to PC-specific death [10]. An IHC analysis of HER2 in 70 metastatic PC tissues showed positive HER2 immunostaining in 64% of samples. While no significant relationship was observed between HER2 overexpression and tumor grade or disease severity and metastases, there was a significant difference (*p* = 0.034) between the average specific survival in patients with HER2 overexpression (33 months) and patients with HER2 negativity (54 months) [11]. Another study analyzed 50 bone metastatic PC cases using IHC and found HER2 overexpression (>1+) in 42% of samples while the PC-specific survival and nonrecurrence rates were significantly lower in the HER2-positive group than in the -negative group (*p* = 0.0084 and *p* = 0.0485, respectively). Furthermore, the PC-specific survival rate after recurrence was significantly higher in the HER2-negative group than in the -positive group (*p* = 0.0247) [12]. Lastly, tissue samples and sera taken from 69 patients with androgen-independent PC treated with docetaxel were prospectively tested for serum HER2 extracellular domain (ECD) by immuno-analysis and tissue expression as determined by IHC and FISH in the tumors. A total of 34.8% of patients had high HER2 ECD (>15 ng/mL). Patients with high HER2 ECD were less responsive (36%) to treatment as measured by prostate-specific antigen (PSA) response compared to low HER2 ECD patients (58%) (*p* = 0.046). Furthermore, HER2 ECD levels were an independent prognostic factor for time to PSA progression [hazard ratio (HR) 2.82; 95% confidence interval (CI) 1.22–6.50; *p* = 0.015] and overall survival (HR 3.24; 95% CI 1.38–7.59; *p* = 0.007). Therefore, high HER2 ECD levels in serum were associated with a worse clinical outcome for patients treated with docetaxel. FISH analysis showed no amplification in all samples [60].

Despite these breakthroughs, HER2 has failed to become an established standard-of-care therapeutic target for PC. A major factor that has contributed to this failure to launch is that in tumors like BC, HER2 overexpression is primarily driven by gene amplification via an increased copy number of the *ERBB2* gene, which is located on chromosome 17 [8,61]. Somatic and germline HER2 missense mutations often lead to *ERBB2* gene amplification [52,62]. Aberrant HER2 mutations and amplifications are commonly discovered in breast tumor genomic profiling, but HER2 overexpression driven by mutations in the diagnosis and prognosis of other cancers has not been fully elucidated. Research groups cannot agree on the prevalence of *ERBB2* gene copy amplification in primary or metastatic PC. Most groups have observed amplification within primary and metastatic PC tissues at very low rates (<10%) [9,52,53,59], but some research groups have found that amplification via gene copy number is absent [9,60,63]. One research group consistently reported low amplification in PC samples at higher rates (40–80%) than most research groups [64,65,66]. Ross and colleagues previously reported HER2 amplification in 44% of 62 PC tumors and 41% of 112 PC samples ranging from TNM stage 2 to 4, respectively [65,66]. Similarly, a study on 44 PC patients demonstrated 53% and 80% low copy amplification in non-metastatic and metastatic samples, respectively, using FISH analysis [64].

We evaluated the genetic alteration status of *ERBB2* across a panel of 32 cancers on the GDC Data Portal website (Figure 2) [67]. We found that the overall alteration frequency was greatest in esophageal cancer (~18%). “Amplifications” of *ERBB2* are the main type of genetic alteration in most cancers followed by “Mutations” and then “Multiple Alterations”. The amplification frequency of *ERRB2* was highest in patients with stomach cancer (SC) followed by esophageal cancer (EC) then BC. PC tumors are evenly divided in the frequency of “Amplifications”, “Mutations”, and “Deep Deletions” of *ERBB2* as the genetic alteration type. Gene alterations overall in PC are very low in comparison to most of the cancers in our pan-cancer analysis (red arrow, <2.5%) (Figure 2). Moreover, this analysis validates previous findings that gene amplification of *ERBB2* in PC is much lower than in SC, EC, and BC (<1%). Our findings support previous observations from other research groups of low to absent prevalence of *ERBB2* gene amplification in PC patients [9,52,53,59,64,65,66].

While the utility of FISH for gene amplification analysis has been successful in BC, the technique is challenging in PC due to the fluorescence signal fading quickly within PC samples, the high cost of the procedure, and the time-consuming nature of the process [8,63]. Clinicians and researchers have primarily used IHC analysis to measure HER2 protein on the surface of PC tissue. Still, it has, unfortunately, demonstrated a divergent range of expression (from 0–100%) due to variations in sample handling, fixation, sample storage, sample staining, and IHC scoring by pathologists [8,9,55,57,68,69]. For example, research groups have used a wide range of HER2 antibodies that include a polyclonal rabbit anti-human HER2 antibody, the monoclonal anti-human HER2 primary antibody TA1, the rabbit anti-peptide antibody pAb1, the AB-3 primary antibody, the TAB 250 murine monoclonal antibody, the HercepTest immunohistochemical kit, or the monoclonal antibody NCL-CB11 detection kit by Ventana [9,10,53,55,56,57,58,60]. Protocols vary greatly for each antibody or kit, which may affect staining intensity within tissue samples and downstream interpretation. The observed variability suggests that standardizing the HER2 antibodies used for detection and clinical application be considered as a potential solution and that HER2 prevalence and its role in PC need further exploration.

Two additional methods of HER2 detection have been identified. Researchers have used loss-of-heterozygosity analysis, which identifies chromosomal changes, to identify several alterations to genes on chromosomes 6, 8, and 13 that are key to PC development [63,70]. However, to the best of our knowledge, no heterozygosity analyses have been investigated or detected in PC at chromosome 17, which is where *ERBB2* is located [71]. Of note, a seminal study used chromogenic in situ hybridization (CISH) on a small sample of PC patient samples to analyze gene amplification and showed that *ERBB2* amplification levels were directly associated with tumor stage, prostate-specific antigen PSA levels, or a high Gleason score in PC patients. Conversely, patients without HER2 amplification lacked a significantly high pathologic stage [63].

While *ERBB2* gene amplification may not drive HER2 protein overexpression in PC, certain patients may still benefit from using anti-HER2 agents. One study reported that even low-level HER2-expressing PC is associated with unfavorable tumor phenotype, rapid disease progression, and poor prognosis [9]. Another recent study modified the upper gastrointestinal tract HER2 scoring system for use in PC diagnosis and reported a high prevalence of HER2 expression in a cohort of predominantly Black men with PC as well as an association between HER2 overexpression and advanced disease [13]. This finding is important as Black men suffer disproportionately from increased incidence and mortality from PC, which is partially attributable to aggressive tumor biology and advanced disease stage at diagnosis [2,3]. Future studies are needed to further validate novel diagnosis and prognosis methods in PC, and to evaluate HER2-targeted therapies. Unfortunately, a current treatment landscape for HER2+ PC does not exist as a standard-of-care option, primarily because of clinical dependence on gene amplification detection by FISH. Continued focus on expanding research studies that thoroughly evaluate the molecular mechanisms that drive PC progression and metastasis, inclusive of HER2 overexpression, leading to lethal outcomes in Black men and other high-risk populations, remains a paramount goal. The ability to target HER2 effectively would expand the therapeutic arsenal of options for HER2+ PC and may offer improved survival outcomes for incurable advanced PC. Additionally, the ability to effectively target HER2 may reduce outcome disparities for Black men who may be more likely to overexpress HER through ancestry-driven mechanisms that remain to be determined.

## 5. HER2, Androgen Receptor, and Other Prostate Cancer Biomarkers

Currently, PSA serves as the most notable PC biomarker and is used as a primary tool for PC screening, disease progression monitoring, and the assessment of therapeutic efficacy [5,72]. Nevertheless, PSA’s specificity for PC is limited, as elevated levels have also been shown to stem from benign conditions such as prostatitis and benign prostatic hyperplasia. Consequently, false-positive results and unnecessary biopsies are common, leading to patient distress, elevated healthcare expenditures, and procedure-associated complications [5,72]. Hence, supplementary metrics like PSA velocity, PSA density, and PSA doubling time as well as imaging biomarkers including multiparametric MRI have been incorporated to augment the specificity and sensitivity of PSA-based PC screening [5,73].

Apart from PSA and other prostate-specific antigens such as prostate acid phosphatase, prostate-specific membrane antigen, and six-transmembrane epithelial antigen of the prostate, there has been increased interest in identifying molecular genetic biomarkers that can also serve as both risk stratification and therapeutic targets [74]. Molecular profiles indicate that increased AR activity is a major contributing factor to PC development [75]. In addition, mutations in the AR ligand-binding domain, along with genetic structural rearrangements, have been implicated in the dysregulation of AR signaling, leading to the development of PC disease subtypes: hormone-sensitive PC and metastatic castration-resistant prostate cancer (mCRPC), suggesting AR mutations may serve as biomarkers predicting sensitivity to AR-related targeted therapies [74,76].

Androgen deprivation therapy, which involves direct inhibition of AR signaling, is the primary therapeutic approach in treating patients with advanced PC disease after the failure of localized treatment [77]. Data have shown that a notable portion of patients undergoing ADT, estimated at 30–50%, eventually advance to mCRPC [74,78]. Poly-ADP ribose polymerase inhibitors (PARPis) have surfaced as a viable treatment option for a subgroup of patients diagnosed with mCRPC who are unresponsive to ADT [79]. PARPis operate via synthetic lethality, leveraging underlying genetic mutations to induce cellular toxicity [79]. Within malignant prostate cells, PARPis initiate synthetic lethality by disrupting the DNA damage response, particularly homologous recombination repair signaling, crucial for maintaining DNA integrity and cellular functionality [79]. Notably, in men with mCRPC and specific homologous recombination response pathway alterations, PARPis demonstrate objective tumor responses and enhance both progression-free and overall survival (OS) rates [80]. Notably, in men with mCRPC and specific homologous recombination response pathway alterations, PARPis demonstrate objective tumor responses and enhance both progression-free and OS rates [80].

In one report, three mCRPC cell lines were analyzed to evaluate the effects of EGFR and HER2 activation on downstream signaling pathways. In vivo experiments with DU145-xenografted mice demonstrated successful tumor response to dual targeting with cetuximab and trastuzumab, particularly in tumors with STAT3 activation. Additionally, the combined treatment reduced tumor-initiating cell capacity, indicating the potential to prevent disease progression. However, PC3 xenografts demonstrated tumor relapse despite treatment, suggesting a mechanism dependent upon alternative sustaining receptors. Findings from this study highlight the therapeutic potential of the dual targeting of EGFR and HER2 with chemotherapy for mCRPC patients, especially those with activated STAT3 or an aggressive HER2+ subtype in PC [81].

HER2 expression has also been evaluated in circulating tumor cells (CTCs) as a prognostic factor in patients with mCRPC [82]. HER2 expression in CTCs was found to be associated with poorer outcomes, including shorter progression-free survival and OS, particularly in patients previously treated with androgen receptor signaling inhibitors. HER2 expression served as an independent prognostic factor for progression-free survival. These findings support HER2 targeting in efforts to identify curative options for more advanced-stage cases of PC.

## 6. Interacting Partners and Networks

To understand the potential role of HER2 in regulating several proteins in the cell, we performed interaction analysis using the PathwayStudio^®^ software [17]. Our data analysis identified *ERBB2* as a direct regulator of key oncogenes such as *MYC* and *BCL2*, and tumor suppressors like *PTEN* in several cancers including PC [83,84,85]. This underscores its central role in cancer biology and provides insights into the intricate molecular networks governing tumorigenesis (Figure 3). The direct regulation of *MYC* by *ERBB2* suggests a mechanism by which *ERBB2* overexpression may promote uncontrolled cell proliferation by driving the transcription of numerous *MYC*-driven genes involved in cell growth and division, thus exacerbating the aggressive nature of tumors [86]. Moreover, *ERBB2*-mediated regulation of *PTEN* indicates a dual mechanism to promote oncogenesis by upregulating survival pathways while simultaneously downregulating tumor suppressive signals [87]. The interplay between *ERBB2* and its direct targets *MYC, PTEN,* and *BCL2* creates a robust network that collaboratively impacts cancer cell biology by promoting proliferative signaling, evading growth suppressors, resisting cell death, and facilitating invasion and metastasis. The potential to disrupt these interactions highlights therapeutic targets to pursue as effective treatments for HER2+ PC tumors.

To gain further insight into the interacting role of HER2/*ERBB2* with other proteins, we generated a “direct” protein–protein interaction network using the previously described method (Figure 4A–C) [17]. In our analysis, we identified critical protein–protein interactions of HER2/*ERBB2* with STAT3, mTOR, and CXCR4. The interaction between HER2/*ERBB2* and STAT3 mediates cell growth and survival through transcriptional regulation, contributing to oncogenesis and therapeutic resistance [88]. Also, the HER2/*ERBB2*-mTOR interaction underscores a convergence of growth factor signaling and nutrient-sensing pathways, emphasizing the importance of mTOR in promoting protein synthesis and cell proliferation [89,90]. Additionally, the HER2/*ERBB2*-CXCR4 interaction plays a crucial role in cancer metastasis, particularly in directing the migration of cancer cells to distant organs [89,91,92]. Mapping these interactions helps to provide a framework of the molecular mechanisms that drive HER2+ cancers and highlights the therapeutic potential of targeting these pathways.

## 7. Lack of Diverse Biospecimens in HER2 Bench Studies

A significant limitation in PC research has been a lack of racially/ethnically diverse and relevant preclinical models with sufficient biological and molecular diversity to reflect the heterogeneity of PC adequately [93,94]. The preponderance of preclinical models disproportionately represents PC in white patients, which is problematic due to the greater negative impact that PC has on Black patients and other racially/ethnically diverse populations [95]. Current models include cell lines, organoids or spheroids, and genetically engineered and patient-derived xenograft (PDX) animal models, which investigate the complex mechanisms driving PC development, disease progression, and treatment resistance [96,97]. Each model has unique advantages and disadvantages, but the disadvantages limit the scope of preclinical application and translational relevance [93,96,98]. The most traditional and widely used in vitro models are three commercially available cell lines and their many derivatives—PC3, LNCaP, and DU145—but hundreds more additional cell lines and sublines have been developed and characterized [94,96]. A PubMed search performed in June of 2024 using the search terms PC3, LNCaP, and DU145 yielded 9783, 10,027, and 2535 references, respectively (Supplementary Data S3). These top three cell lines represent metastatic disease derived from white patients [96]. Several cell lines generated from the PDXs of primary and metastatic PC taken from white men are also commercially available [96]. By comparison, only a single commercially available cell line representing metastatic disease derived from a Black patient (MDA-PCa-2b) exists [96]. Of note, recent genetic ancestry analysis of the commercially available cell line, E006AA-hT, which was previously reported as a primary PC cell line derived from a Black patient, showed that it carried 91.3% European American ancestry and was, therefore, misclassified as a Black cell line in PC research [96,99]. Not one commercially available cell line that represents Asian, Hispanic, or Native American patients exists [93,96]. The novel cell lines, RC77-T/E and KUCaP, represent primary PC derived from a Black patient and metastatic PC derived from an Asian patient, respectively; however, these are not commercially available [96].

The more modern preclinical models—organoids and PDXs—have revolutionized the study of PC; however, the limited availability of diverse patient samples and insufficient tumor growth in immunodeficient mice, among other confounders, make diverse spheroids and PDXs challenging to establish [96]. Despite this, a few research groups have successfully established PDXs representing minoritized populations, including the MD Anderson PC PDX series derived from 47 racially diverse patients [100,101,102,103,104]. The National Cancer Institute (NCI) has also developed a national repository of patient-derived models that includes diverse organoids and PDXs and their associated datasets, to increase biological and clinical diversity in PC research [105,106]. Furthermore, multiple research groups have developed racially diverse organoids and PDXs grown in suspension culture over the years [107,108,109,110]. However, variations in the protocols and analysis methods among research groups can limit translational application and clinical relevance. Despite the generation of over 300 known PDXs to date, increased sample diversity and access still need to be addressed. The role of PDXs in preclinical PC research will continue to grow and prioritizing increased diversity and access would reduce the current health disparities experienced by Black men and other high-risk populations in the long term.

## 8. Lack of Diversity in HER2 Clinical Trials

Without adequate diverse representation and inclusion in clinical trials, advances in medical innovation may, in fact, exacerbate existing inequities in PC outcomes due to differences in access to precision medicine. To address this challenge, we must continue efforts to diversify biospecimens for bench science and increase diverse enrollment of study participants into PC clinical trials. A prime example highlighting the importance of these efforts includes the targeting of HER2 in the past. A decade ago, phase II clinical trials targeting HER2 with anti-HER2 drugs in PC patients were initiated but failed due to 1) a lack of study participants with HER2+ tumors and 2) poor efficacy with anti-HER2 drugs including trastuzumab and lapatinib as single agents [111,112,113,114,115]. Upon completion, the authors suggested increased enrollment of HER2+ patients to better evaluate the ability of anti-HER2 drugs to improve PC outcomes. In the previous studies, PC patients were not stratified by race, and racial differences in HER2 expression were not evaluated. The inclusion of diverse patients who may have increased levels of HER2 overexpression in similarly designed studies may reveal differences and improvements in treatment response.

Because most patients evaluated in PC bench studies and clinical trials are of European descent, genetic indicators that steer molecular mechanisms that aggravate outcome disparities for high-risk populations of non-European descent are largely missed. This reality is counterintuitive as the highest-risk Black population are most likely to die from PC, but less likely to be included in key studies. A few studies have recently implemented targeted accrual of Black men to PC clinical trials and have revealed better treatment response and increased OS in Black men compared to white men receiving PC drug treatments for advanced disease, highlighting the potential to decrease the mortality disparity with targeted therapeutic options optimized for this high-risk population [116,117]. In the PROCEED study, Black men with mCRPC treated with sipuleucel-T had significantly improved OS vs. white men with mCRPC treated with sipulecuel-T [116]. After a median follow-up of 46.6 months, median OS was 35.2 months for Black men and 29.9 months for white men: HR 0.81, 95% CI 0.68–0.97; *p* = 0.03. Along with other known prognostic factors, self-reported Black race was independently associated with prolonged OS [116]. In a separate study, despite worse performance status, higher testosterone, higher PSA, and lower hemoglobin in Black study participants with mCRPC (*n* = 500) compared to white study participants with mCRPC (*n* = 7528), the median OS was 21.0 months in Black men (95% CI, 19.4 to 22.5 months) vs. 21.2 months in white men (95% CI, 20.8 to 21.7 months) [117]. A pooled multivariable hazard ratio of 0.81 (95% CI, 0.72 to 0.91) demonstrated that Black men had an overall statistically significant decreased risk of death compared to white men (*p* < 0.001) [117]. By including adequate representation of Black men in PC research studies and clinical trials such as these, the benefits of precision medicine will be equitable for all PC patients.

## 9. Conclusions

In this review, we have, for the first time, shed light on the role of HER2 in PC through a racially inclusive lens. HER2 overexpression has been evaluated over the past decade in homogenic studies that have implicated its association with therapy resistance and worse outcomes. However, progress has been stunted and results are inconclusive as Black men with PC who may have a higher prevalence of HER2 overexpression have not been included. We intend to inspire other groups to consider the inclusion of diverse biospecimens and the recruitment and enrollment of diverse patients to studies that continue to investigate HER2 in PC.

## Data Availability

The data supporting the conclusions of this review article will be made available by the authors on request.

## Supplementary Materials

The following supporting information related to Figure 1, Figure 3, and Figure 4 can be downloaded at: https://www.mdpi.com/article/10.3390/cancers16193262/s1, Supplementary Data S1, Supplementary Data S2, and Supplementary Data S3. These supplementary data indicate gene interaction, entity function and references that were used to build the relationship.
